# MytiLec, a Mussel R-Type Lectin, Interacts with Surface Glycan Gb3 on Burkitt’s Lymphoma Cells to Trigger Apoptosis through Multiple Pathways

**DOI:** 10.3390/md13127071

**Published:** 2015-12-12

**Authors:** Imtiaj Hasan, Shigeki Sugawara, Yuki Fujii, Yasuhiro Koide, Daiki Terada, Naoya Iimura, Toshiyuki Fujiwara, Keisuke G. Takahashi, Nobuhiko Kojima, Sultana Rajia, Sarkar M. A. Kawsar, Robert A. Kanaly, Hideho Uchiyama, Masahiro Hosono, Yukiko Ogawa, Hideaki Fujita, Jiharu Hamako, Taei Matsui, Yasuhiro Ozeki

**Affiliations:** 1 Department of Life and Environmental System Science, Graduate School of NanoBio Sciences, Yokohama City University, 22-2 Seto, Kanazawa-ku, Yokohama 236-0027, Japan; hasanimtiaj@yahoo.co.uk (I.H.); yasukoide04@yahoo.co.jp (Y.K.); akht.51.314@gmail.com (N.I); nobuhiko@yokohama-cu.ac.jp (N.K.); rajia_bio@yahoo.com (S.R.); akawsarabe@yahoo.com (S.M.A.K.); kanaly@yokohama-cu.ac.jp (R.A.K.); hideho@yokohama-cu.ac.jp (H.U.); 2 Department of Biochemistry and Molecular Biology, Faculty of Science, University of Rajshahi, Rajshahi-6205, Bangladesh; 3 Division of Cell Recognition Study, Institute of Molecular Biomembrane and Glycobiology, Tohoku Pharmaceutical University, 4-4-1 Komatsushima, Aoba-ku, Sendai 981-8558, Japan; ssuga@tohoku-pharm.ac.jp (S.S.); mhosono@tohoku-pharm.ac.jp (M.H.); 4 Department of Pharmacy, Faculty of Pharmaceutical Science, Nagasaki International University, 2825-7 Huis Ten Bosch, Sasebo, Nagasaki 859-3298, Japan; yfujii@niu.ac.jp (Y.F.); fujiwara@niu.ac.jp (T.F.) yogawa@niu.ac.jp (Y.O.); fujita@niu.ac.jp (H.F.); 5 Graduate School of Medical Life Science, Yokohama City University, 1-7-29 Suehiro-cho, Tsurumi-ku, Yokohama 230-0045, Japan; daiki.tera@tsurumi.yokohama-cu.ac.jp; 6 Graduate School of Agricultural Science, Tohoku University, 1-1 Amamiya-machi, Tsutsumidori, Aoba-ku, Sendai 230-0045, Japan; keisuke.takahashi.b3@tohoku.ac.jp; 7 Department of Natural Science, Varendra University, Rajshahi-6204, Bangladesh; 8 Department of Chemistry, Faculty of Sciences, University of Chittagong, Chittagong-4331, Bangladesh; 9 Department of Biology, School of Health Sciences, Fujita Health University, Toyoake, Aichi 470-1192, Japan; jhamako@fujita-hu.ac.jp (J.H.); tmatsui@fujita-hu.ac.jp (T.M.)

**Keywords:** Burkitt’s lymphoma cells, caspase-9/3, globotriose (Gb3), JNK, *Mytilus galloprovincialis*, MEK/ERK, MytiLec, p21: p38 kinase, R-type lectin, TNF-α, β-trefoil

## Abstract

MytiLec; a novel lectin isolated from the Mediterranean mussel (*Mytilus galloprovincialis*); shows strong binding affinity to globotriose (Gb3: Galα1-4Galβ1-4Glc). MytiLec revealed β-trefoil folding as also found in the ricin B-subunit type (R-type) lectin family, although the amino acid sequences were quite different. Classification of R-type lectin family members therefore needs to be based on conformation as well as on primary structure. MytiLec specifically killed Burkitt's lymphoma Ramos cells, which express Gb3. Fluorescein-labeling assay revealed that MytiLec was incorporated inside the cells. MytiLec treatment of Ramos cells resulted in activation of both classical MAPK/ extracellular signal-regulated kinase and extracellular signal-regulated kinase (MEK-ERK) and stress-activated (p38 kinase and JNK) Mitogen-activated protein kinases (MAPK) pathways. In the cells, MytiLec treatment triggered expression of tumor necrosis factor (TNF)-α (a ligand of death receptor-dependent apoptosis) and activation of mitochondria-controlling caspase-9 (initiator caspase) and caspase-3 (activator caspase). Experiments using the specific MEK inhibitor U0126 showed that MytiLec-induced phosphorylation of the MEK-ERK pathway up-regulated expression of the cyclin-dependent kinase inhibitor p21, leading to cell cycle arrest and TNF-α production. Activation of caspase-3 by MytiLec appeared to be regulated by multiple different pathways. Our findings, taken together, indicate that the novel R-type lectin MytiLec initiates programmed cell death of Burkitt’s lymphoma cells through multiple pathways (MAPK cascade, death receptor signaling; caspase activation) based on interaction of the lectin with Gb3-containing glycosphingolipid-enriched microdomains on the cell surface.

## 1. Introduction

During the present decade, genome databases have been established for numerous mollusk species, and are very useful in industrial and ecological studies [1]. “MytiBase” is an impacted EST (expressed sequence tags) database for the Mediterranean mussel, *Mytilus galloprovincialis*, a worldwide species that plays major roles in marine ecosystems and is commercially important [2,3]. Recent advances in mussel genomics have elucidated the crucial genes in various physiological processes, and shown that certain signal transduction molecules that regulate innate immunity in mussels have analogues in higher animals [4,5,6]. Recent studies have focused on such genes that are capable of being activated by various environmental pollutants. Progress in this area will have important ecological, economic, and human health implications.

Mussels have been found to produce several interesting bioactive compounds. A furan fatty acid isolated from the green-lipped mussel (*Perna canaliculus*) suppresses inflammatory processes [7] and breast cancer cell growth *in vitro* [8], and is currently undergoing clinical trials [8,9]. Macromolecules isolated from mussels or other bivalves are useful in food science and as research tools in life sciences [10].

A major focus in glycobiology is the regulation of living systems through molecular interactions between glycans (polysaccharides) and their receptors, which include glycan-binding proteins termed "lectins". Monosaccharides comprising a glycan chain are arranged according to axial or equatorial configuration of hydroxyl groups around each carbon atom. The glycosidic linkages are connections between anomeric carbons of adjacent monosaccharides. Changes in length and branching of glycan chains occur during disease processes, ontogenic development, differentiation, and regeneration [11]. Changes in somatic cells that lead to cancer or reprogramming are often associated with striking changes in glycan chains of glycoproteins and glycosphingolipids to an immature or ancestral form similar to glycans found in Golgi bodies or lower organisms [11,12].

MytiLec is an α-Gal-binding lectin that we isolated in 2012 from the Mediterranean mussel *Mytilus galloprovincialis* (family Mytilidae). Its primary structure is a 17 kDa polypeptide (149 amino acids, including one Trp and no Cys) containing triple tandem-repeating 50-a.a. subdomains [13,14]. The cDNA sequence coding MytiLec has also been deposited in the MytiBase EST library [15]. Deduced a.a. from cDNA coding a Gal/GalNAc-binding lectin isolated from another mytilid mussel, *Crenomytilus grayanus*, gives similar primary structure [16,17]. We showed (by frontal affinity chromatography) that MytiLec binds specifically to globotriose (Gb3; Galα1-4Galβ1-4Glc) and is toxic to Burkitt’s lymphoma cells (which express globotriaosylceramide-containing Gb3 glycan on the cell surface) [13].

Nevertheless, the primary structure of MytiLec has been quite novel, its 3-D structure obtained by crystallography analysis of MytiLec identified that the lectin had a β-trefoil folding (PDB entry 3wmu at 1.1 Å and PDB entry 3wmv at 1.05 Å [18] that is found in the B-subunit of ricin [19], a representative plant toxin isolated from castor beans (*Ricinus communis*). Lectins having the β-trefoil folding and similar primary structures with the B-subunit of ricin are included in the “R-type lectin family”. In addition to possessing this characteristic folding, MytiLec was a non-covalently bound dimer consisting of two polypeptides having the glycan-binding activity in each sub-domain (Jeremy R.H. Tame, personal communication). Six carbohydrate-binding sites of dimer MytiLec was essential for the cytotoxicity (data not shown) similar to the property of another cytotoxic R-type lectin isolated from mushroom *Clitocybe nebularis* [20]. Taken together, MytiLec fits in as a new member of the R-type lectin family.

Some R-type lectins have additional domains as toxic subunits. Pierisin, isolated from *Pieris rapae* (cabbage butterfly), has an ADP-ribosyltransferase domain in the polypeptide and three R-type lectin domains. Pierisin induces apoptosis in HeLa cells by binding to surface Gb3 and Gb4 (GalNAcβ1-3Galα1-4Galβ1-4Glc) glycans [21]. In addition to the original MytiLec, two MytiLec variants (termed MytiLec2 and MytiLec3) containing a pore-forming aerolysin [22]-like domain in the polypeptide that creates pores into infectious organisms and kills them through initiation of innate immunity, according to the recently updated MytiBase [4].

MytiLec does not have additional functional domains or subunits beside glycan-binding domains, in contrast to other R-type lectins, although it is capable of inducing cytotoxicity. It thus occupies a unique category within the R-type lectin family. The mechanisms whereby MytiLec transmits its signals through cells to activate various signal transduction molecules for induction of cancer cell apoptosis are of great interest. We used experimental cell line, Ramos with high levels of Gb3 expression to study apoptosis-inducing molecules (mitogen-activated protein kinases (MAPK) cascade, mitochondria-controlling caspase, and death receptor signal) activated by MytiLec in Burkitt’s lymphoma cells.

## 2. Results and Discussion

### 2.1. Glycan-Binding and Cell Agglutination of Recombinant MytiLec

**Figure 1:**
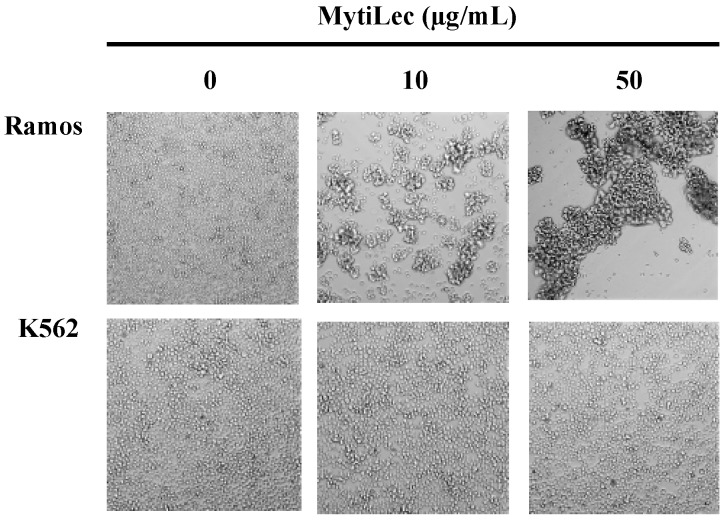
Different cell agglutination activities of MytiLec. MytiLec (0, 10, and 50 μg/mL) was applied to Ramos (5 × 10^5^ cells) and K562 (2 × 10^5^ cells) cells and observed by phase contrast microscopy.

MytiLec agglutinated Burkitt’s lymphoma-derived Ramos cells (high Gb3 expression) [23] but did not agglutinate K562 erythroleukemia cells (no Gb3 expression). Strong agglutination was observed for Ramos, as revealed by large cell masses (Figure 1).

### 2.2. Cytotoxic Effects of MytiLec on Burkitt’s Lymphoma Cell Lines

**Figure 2:**
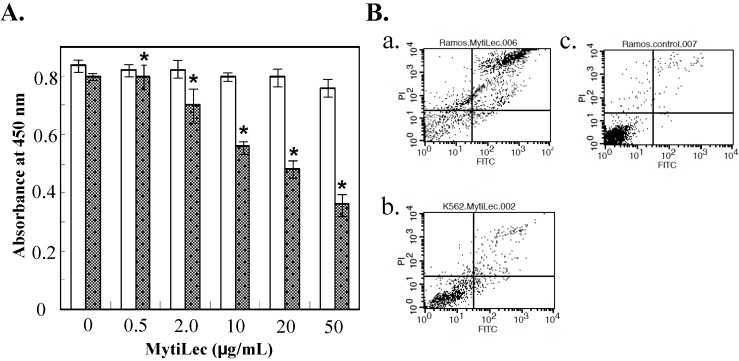
Reduction of cell viability by MytiLec. (**A**) Determination of viability by WST-8 assay. Dotted columns: Ramos. White columns: K562. Cells (2 × 10^5^ of Ramos; 5 × 10^5^ of K562) were incubated with various MytiLec concentrations as shown. Error bars: SE (*n* = 3); (**B**) Annexin V-binding and propidium iodide (PI) incorporation in MytiLec-treated cells. Horizontal axis: binding of FITC-labeled annexin V. Phosphatidylserine externalization and PI incorporation were evaluated by FACS analysis using MEBCYTO apoptosis kit. Ramos (**a**,**c**) and K562 (**b**) cells were treated with MytiLec (**a**,**b**: 20 μg/mL; **c**: 0 μg/mL) for 30 min at 4 °C. Data shown are mean values with error bars = SD of triplicate experiments. Asterisks = significant differences (*p* < 0.05) between treated and control groups.

Cytotoxic effects of MytiLec administration were evaluated by WST-8 assay rather than trypan blue assay because agglutinated cell masses were not effectively stained by trypan blue reagent. Ramos and K562 cells were cultured for 24 h, treated with MytiLec, and reduction in proportion of living cells was assayed by measuring absorbance at 450 nm. Viability was reduced in comparison with control (nontreated) cells for Ramos treated with 10 μg/mL of MytiLec, indicating a cytotoxic effect. Viability of K562 cells, which do not express Gb3, was unaffected by MytiLec treatment (Figure 2A).

**Figure 3:**
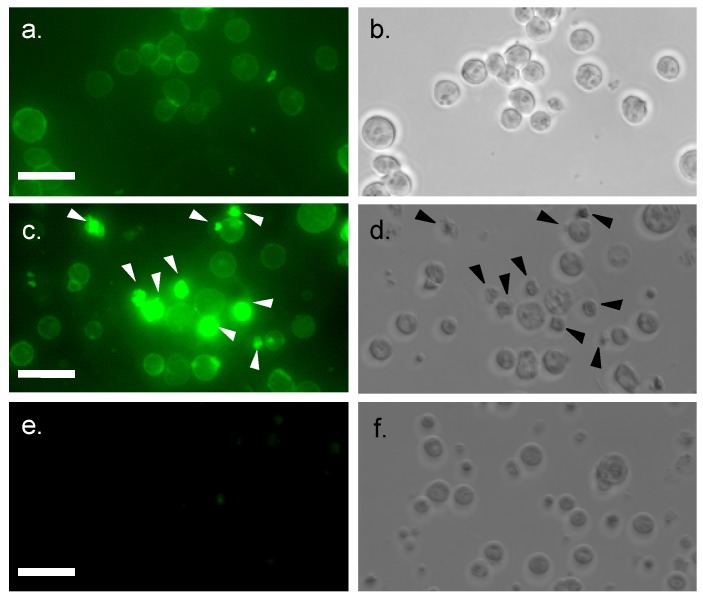
Internalization of FITC-conjugated MytiLec into Burkitt’s lymphoma cells. Incubation time: 0 min (**a**,**b**); and 2 h (**c**–**f**). Cells are treated with FITC-MytiLec in the presence of 25 mM D-galactose, as negative control (**e**,**f**). Cells were observed by fluorescence (**a**,**c**,**e**; ex 498 nm and em 522 nm) and phase-contrast (**b**,**d**,**f**), respectively. Arrows in c and d indicated shrunken cells. Bars indicated 50 μm.

Fluorescence activated cell sorting (FACS) analysis revealed that MytiLec treatment led to deleterious biological processes such as cell membrane inversion and loss of membrane integrity. Horizontal axes in Figure 3B show binding of Fluorescein isothiocyanate (FITC)-labeled annexin V, and vertical axes show incorporation of propidium iodide. Increasing MytiLec concentration was associated with shifting of annexin V-positive and propidium iodate-positive populations into the right and upper portions (respectively) of these histograms. K562 cells were unaffected by MytiLec treatment. The membrane inversion and penetration observed in MytiLec-treated Ramos cells were consistent with results of our previous study on Raji cells, another Burkitt's lymphoma cell line [13]. These effects on the Ramos cell membrane (Figure 2B) appeared to be associated with the cytotoxic effect of MytiLec (Figure 3). MytiLec may increase cell fragility by suppressing biosynthesis of cell surface membrane proteins. The triggering concentration of MytiLec is lower for apoptosis (~10 μg/mL) than for necrosis (>20 μg/mL). These observations may be related to the functions of MytiLec in caspase activation and TNF-α production (Section 2.4).

### 2.3. Internalization of MytiLec into Burkitt’s Lymphoma Cells

Internalization of fluorescein-conjugated MytiLec (20 μg/mL) by Burkitt's lymphoma cells was demonstrated by confocal microscopy. Cell surface fluorescence was observed at the beginning (Figure 3a) and after 2 h incubation, strong intracellular fluorescence was detected due to the migration of FITC-MytiLec (Figure 3c *vs.* d). Such internalization was similar to that observed for TF-antigen-binding BEL lectin [24], an R-type lectin purified from mushroom. Cells with internalized MytiLec looked shrunken and irregular shaped with a characteristic rough surface (Figure 3c,d, arrows). This internalization was totally inhibited by the co-presence of d-galactose, a haptenic sugar of the lectin (Figure 3e). It can be assumed that the internalization of MytiLec activated a number of cell signaling pathways (described in following sections) whether inhibition of this internalization by the sugar gives an idea about the mechanism of Gb3-dependent signaling.

### 2.4. Activation of MAPK Pathways by MytiLec

MAPKs play essential roles in cell growth and differentiation, cell cycle, and cell death. We found that MytiLec activates several MAPK pathways in Burkitt’s lymphoma cells.

#### 2.4.1. Activation of MEK-ERK Pathway

**Figure 4:**
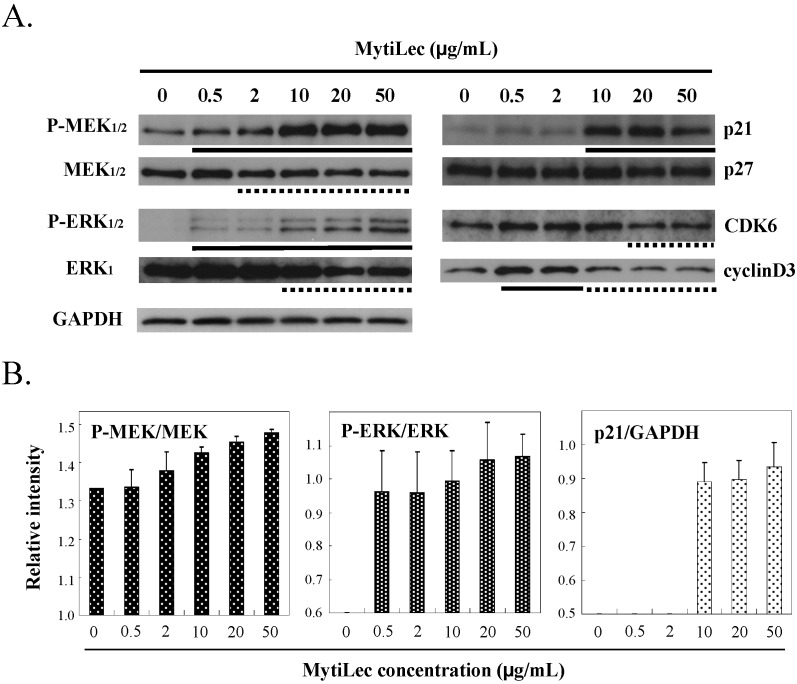
Effects of MytiLec treatment on MEK, ERK, and cell cycle-related molecules in Burkitt’s lymphoma Ramos cells. (**A**) Phosphorylation and expression levels of MEK1/2, ERK1/2 and p21, p27, CDK6 and cyclinD3 were shown, respectively. Cells (4 × 10^5^ in each experiment) were treated with various concentrations of MytiLec as shown, and activation levels were evaluated by Western blotting of lysates. Solid and dotted lines indicated increasing and decreasing trends, respectively. GAPDH: Glyceraldehyde 3-phosphate dehydrogenase; (**B**) Relative densitometric quantification of P-MEK/MEK, P-ERK/ERK and p21/GAPDH. Each experiment was repeated three times.

In Ramos, MytiLec activated the classical MAPK pathway of MAPK/extracellular signal-regulated kinase (MEK)_1/2_ and extracellular signal-regulated kinase (ERK)_1/2_ signaling cascade in a dose-dependent manner, as shown by Western-blotting (Figure 4A, P-MEK_1/2_ *vs.* MEK_1/2_ and P-ERK_1/2_ *vs.* ERK_1_). No such phosphorylation occurred in K562 (data not shown). Phosphorylation of the MEK-ERK pathway by MytiLec resulted in expression of the cyclin-dependent kinase (CDK) inhibitors p21 (Figure 4 column p21). In contrast, up-regulated levels of CDK6 and cyclinD3 were slightly reduced by MytiLec (Figure 4).

Expression of p21 (which binds to CDK and inhibits its activity) is enhanced by various external stimuli and stress factors, resulting in cell cycle arrest at the G_0/1_ phase [25]. Up-regulation of p21 level by MEK has been documented using specific inhibitors [26]. The above findings indicate that binding of MytiLec and Gb3 on the cells induced MEK-ERK pathway activation and p21 expression, eventually resulting in cell cycle arrest.

#### 2.4.2. Phosphorylation of Stress-Activated MAPK Pathways (JNK, p38 Kinase)

**Figure 5:**
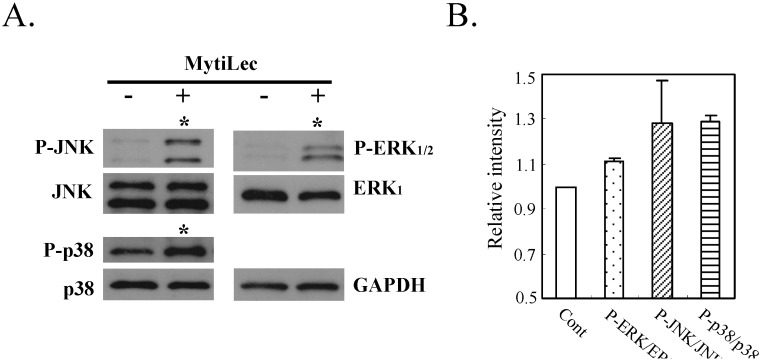
Phosphorylation of JNK and p38 kinase by MytiLec in Burkitt's lymphoma Ramos. Cells (5 × 10^5^) were treated with (+) or without (−) 20 μg/mL MytiLec, and phosphorylation was evaluated by Western-blotting of cell lysates. (**A**) P-ERK, P-JNK, and P-p38: phosphorylated forms of ERK, JNK, and p38 kinase, respectively. Asterisks: increased phosphorylation; (**B**) Relative densitometric quantification of P-ERK/ERK, P-JNK/JNK and P-p38/p38. Each experiment was repeated three times.

In addition to the classical MAPK pathway (MEK-ERK), MytiLec phosphorylated stress-activated MAPK pathways [c-Jun *N*-terminal kinase (JNK) and p38 kinase] in Ramos (Figure 5, asterisks). Evidently, ERK mediated transduction of the MytiLec/ Gb3 binding signal to JNK and p38 kinase. The MytiLec-generated stimuli were equivalent to signals generated by oxidative stresses [27]. A recent study indicates that the MEK/ERK pathway itself can activate both JNK and p38 kinase [28]. Certain mannose-binding proteins inhibited cell proliferation through activation of these pathways [29,30]. Stress-activated kinases play roles in tumor suppression, apoptosis, termination of cell differentiation, and autophagy [31].

#### 2.4.3. MytiLec-Induced Phosphorylation of MEK-ERK Pathway Causes Cell Cycle Arrest through p21 Up-Regulation

**Figure 6:**
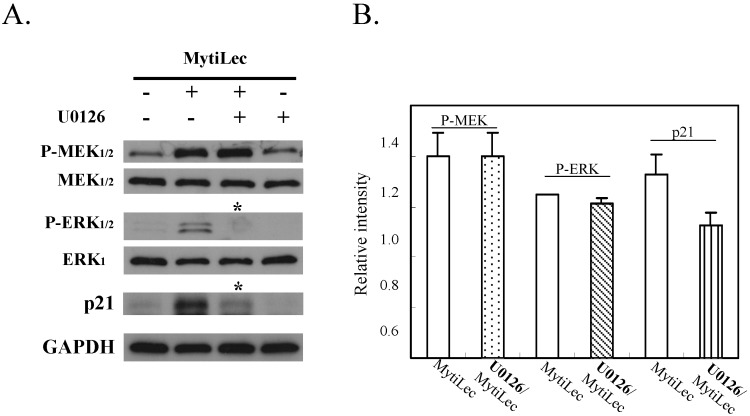
Treatment with MEK inhibitor U0126 reversed up-regulation of p21 expression mediated by MytiLec-induced phosphorylation of MEK-ERK pathway. (**A**) Burkitt's lymphoma Ramos cells (4 × 10^5^) were treated with (+) or without (−) 20 μM U0126 for 5 h, and then with 20 μg/mL MytiLec. Expression levels of P-MEK_1/2_, P-ERK_1/2_, and p21 were evaluated by Western-blotting. Asterisks: disappearance of signals; (**B**) Relative densitometric quantification of P-MEK, P-ERK and p21 with (U0126/MytiLec) or without (MytiLec) the MEK inhibitor. Each experiment was repeated three times.

Pre-incubation with U0126 (a specific MEK_1/2_ inhibitor) significantly reduced MytiLec-induced phosphorylation of ERK_1/2_ in Ramos (Figure 6, P-ERK_1/2_ *vs.* ERK_1_). U0126 also reversed MytiLec-induced enhancement of p21 expression (Figure 6, p21, asterisks). U0126 had no effect on expression of MEK or P-MEK (Figure 6, P-MEK_1/2_ *vs.* MEK_1/2_), consistent with the finding that U0126 directly inhibits the downstream pathway under P-MEK [32].

In conclusion, MytiLec/Gb3 binding in Ramos promoted both the classical MAPK pathway (MEK-ERK) and stress-activated MAPK pathways (JNK, P38 kinase). MytiLec-induced phosphorylation of the MEK-ERK pathway resulted in up-regulation of p21 expression that might lead to cell cycle arrest.

### 2.5. TNF-α Induction and Caspase Activation in Ramos Cells

**Figure 7:**
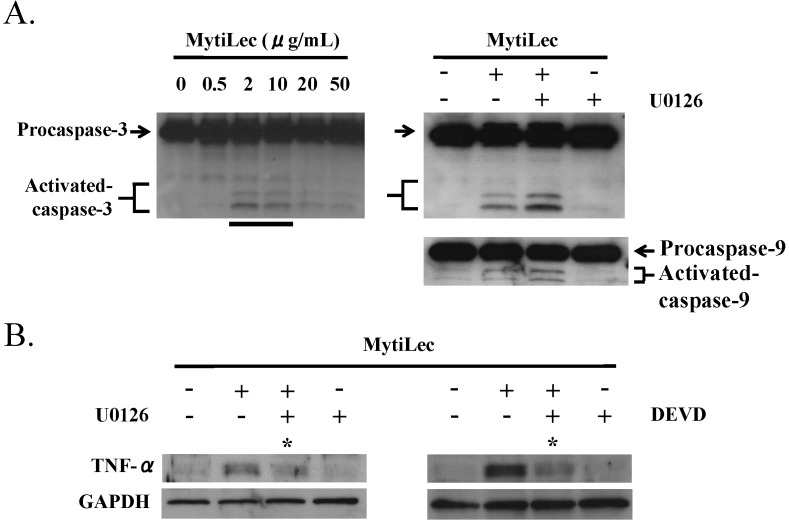
Activation by MytiLec of procaspase-3, procaspase-9, and TNF-α. (**A**) Activation of procaspase-3 in Ramos (4 × 10^5^ cells) incubated with various concentrations of MytiLec as shown, evaluated by Western blotting; (**B**) Up-regulation of TNF-α by MytiLec, and inhibition of caspase activation and TNF-α expression by P-MEK inhibitor U0126 and caspase-3 inhibitor Zn-DEVD-FMK (DEVD). Ramos (4 × 10^5^ cells) were treated with (+) or without (−) 10 μM U0126 or 10 μM DEVD for 2 h, and then with 20 μg/mL MytiLec. TNF-α expression and caspase-3/-9 activation were evaluated by Western blotting. Solid and dotted lines indicated increasing and decreasing trends of phosphorylation, respectively. Asterisks: disappearance of signals. Each experiment was repeated three times.

MytiLec treatment of Burkitt’s lymphoma Ramos cells triggered production of tumor necrosis factor (TNF)-α and activation of caspase-9 and caspase-3 (Figure 7A). The up-regulation of TNF-α by MytiLec was inhibited by P-MEK inhibitor U0126 and by caspase-3 inhibitor Zn-DEVD-FMK (Figure 7B). Association of TNF-α induction with caspase-3 activation was consistent with a previous observation by Burguillos *et al.* [33], and suggests that TNF-α expression is concurrently regulated by the MEK-ERK pathway and caspase-3 activation.

To elucidate the caspase activation pathway, U0126 was applied to Ramos prior to MytiLec administration. Neither caspase-9 nor caspase-3 was inhibited by U0126, indicating that MytiLec-induced activation of caspase-9 and -3 in these cells is independent of the MEK-ERK pathway (Figure 7A, MytiLec(+)/U0126(+)).

Previous studies have demonstrated stimulation via phosphorylation of various signal transduction molecules by lectins isolated from lower organisms. In mouse macrophage cell lines, a recombinant B-subunit of ricin was shown to stimulate signal transduction pathways through production of inducible nitric oxide synthase, TNF-α, and interleukin-6 [34]. Human TNF-α reduced the phagocytic ability of mussel hemocytes [35]. The normal endogenous role of MytiLec in *M. galloprovincialis* remains unclear. Genome database analysis suggests that it may function in innate immunity [4]. Certain signal transduction molecules in *M. galloprovincialis* are also found in vertebrates (including humans) [5,36], suggesting similarities in the fundamental regulatory mechanisms of growth, differentiation, and cell proliferation. A role of native MytiLec in supporting innate immunity could explain its cytotoxic activity against Burkitt's lymphoma cells, since the mussel and vertebrate cells may have common surface glycans such as Gb3, as well as similar cell regulatory mechanisms. Along this line, we are attempting to identify the endogenous ligands of Gb3 and related glycans in the mussel. We previously observed binding of MytiLec to endogenous glycans in mussel tissue [13]. Surface Gb3 expression was observed on cultured cells derived from sea bass (*Dicentrarchus labrax*) [37].

There are difficulties in studying marine drugs based on proteins. Proteins trigger immune responses, and present logistical research problems because of their large size. However, recent studies based on each of sialic acid-binding and α-galacotside-binding lectins-coding genes from fish and sea urchin, respectively successfully recombined the genes into an adenovirus vector and applied the lectins for oncotherapy *in vitro* [38,39]. MytiLec, with its novel cytotoxic properties, has great potential for similar therapeutic application through Gb3-signaling.

Ponting *et al.* [40] reviewed studies since 1990 of β-trefoil folding, including the question of why toxins (ricin B-chain [19]), cytokines (fibroblast growth factor [41], interleukin-1 [42]), enzymes (non-catalytic domain of glycosyltransferases [43]) and protease inhibitors (Kunitz-type protease inhibitor [44]) are synchronically assigned to the same 3D structural group even though their primary structures have low similarity (<12%). The polypeptides are highly conserved, with triple-tandem repeating sequences that contain four β-sheets and one α-helix in each subdomain. It appears that β-trefoil folding is one of the most versatile templates for protein conformation, and is involved in a wide range of physiological functions in many organisms. The primary structure of MytiLec is unique in that gene coding for R-type lectins has not been reported for any other *Mytilus* species. We hypothesize that an ancestral gene was synchronically modified for β-trefoil folding in MytiLec in association with its role in innate immunity.

The cell regulatory properties of Gb3-binding lectins are clearly diverse and merit further study. SAL, a Gb3-specific SUEL/RBL-type lectin isolated from catfish (*Silurus asotus*) eggs, phosphorylated several signal transduction molecules, resulting in cell cycle delay but had no cytotoxic effect [45], unlike MytiLec. SAL was found to down-regulate the multidrug resistance (MDR) 1 P-glycoprotein (MDR1 P-gp) on Burkitt's lymphoma cells through Gb3 and did not directly influence the viability of the cells. In this study, Gb3 was found to be an effective trigger to regulate cancer cell growth through apoptosis. Therefore, along with Burkitt’s lymphoma cells, a number of Gb3-expressing cancer cell lines [46] like HeLa, MCF-7 and T47D, can be good targets to study cell signaling. In particular, detailed investigation of these lectins and their properties will be useful for development of novel anti-cancer drugs and therapeutic strategies.

## 3. Experimental Design

### 3.1. Preparation of MytiLec

MytiLec was purified from Mediterranean mussel *M. galloprovincialis* according to our previous report [13].

### 3.2. Cell Lines and Culture

Burkitt’s lymphoma Ramos cell line was obtained from American Type Culture Collection (CRL-1596). Erythroleukemia K562 cell line were obtained from the Cell Resource Center of Biomedical Research, Institute of Development, Aging and Cancer, Tohoku University (Sendai, Japan). Cells were cultured in RPMI-1640 medium (Nissui Pharmaceutical, Tokyo, Japan) supplemented with 10% FBS and antibiotic-antimycotic solution (Life Technologies, Carlsbad, CA, USA), and maintained at 37 °C in a 95% air/5% CO_2_ atmosphere.

### 3.3. Cytotoxicity and Cell Viability Assays

Cells were maintained in RPMI-1640 supplemented with heat-inactivated FBS (10% *v*/*v*), penicillin (100 IU/mL), and streptomycin (100 μg/mL) at 37 °C in a 95% air/5% CO_2_ atmosphere. Cytotoxic activity and cell growth following treatment with various concentrations of SAL (0–100 μg/mL) were determined using Cell Counting Kit-8 containing WST-8 (Dojindo Laboratories, Kumamoto, Japan) [47]. To evaluate the inhibitory effects of sugars, sucrose and melibiose (each 200 mM) were co-incubated with SAL (100 μg/mL) for 24 h and applied to the assay system. Cells (5 × 10^4^, in 90 µL solution) were seeded into a 96-well flat-bottom plate and treated with various concentrations of MytiLec (10 µL) for 24 h at 37 °C. The effect of MytiLec on cell growth was assayed by addition of WST-8 solution (10 µL) to each well and incubation for 4 h at 37 °C. The reduction in proportion of living cells was assayed by measurement of absorbance at 450 nm using a GloMax Multi Detection System (Promega, Madison, WI, USA).

### 3.4. Fluorescein Isothiocyanate (FITC)-Conjugated MytiLec

For confocal microscopy, purified MytiLec (2 mg) was chemically conjugated with NH_2_-reactive fluorescein isothioycanate (Dojindo Laboratories, Kumamoto, Japan) according to the manufacturer’s protocol. Burkitt’s lymphoma cells (100 μL; 2 × 10^5^ cells/mL) in RPMI-1640 (Sigma-Aldrich, St. Louis, MO, USA) supplemented with 10% FBS were seeded onto 18-mm round cover slips in Petri dishes and left to attach overnight at 37 °C in a humidified 5% CO_2_ atmosphere. The cover slips were washed three times with 2 mL PBS, incubated for 2 h with 50 μg/mL FITC-labeled lectin in PBS, and washed again. Cells were fixed with 4% paraformaldehyde for 15 min. Images at various focal planes were taken with a Leica TCS PS5 confocal microscope. 498 nm lasers were used for excitation of FITC.

### 3.5. Protein Expression of Signal Transduction Molecules and Their Phosphorylated Forms

Cells (5 × 10^5^) were cultured for 12 or 24 h in RPMI-1640 with or without MytiLec (100 µg/mL) at 37 °C in 95% air/ 5% CO_2_. Cell lysate was prepared using an AllPrep RNA/Protein Kit (Qiagen, Hilden, Germany), subjected to SDS-PAGE (12.5% separation gel), and electrotransferred onto polyvinylidene difluoride (PVDF) membrane (pore size 0.45 µm) (Hybond-P; GE Healthcare Bio-Sciences AB, Uppsala, Sweden) according to the manufacturer’s protocol. The membrane was treated with blocking buffer (Blocking One; Nacalai Tesque, Kyoto, Japan) for 1 h at room temperature and washed with Tris-buffered saline (TBS) containing 0.05% Tween-20. The primary antibodies used were directed to p21 Waf1/Cip1 (1:1000, rabbit mAb, clone 12D1; Cell Signaling Technology, Danvers, MA, USA (CST)), p27 Kip1 (1:2000, rabbit mAb; clone D69C12; CST), CDK6 (1:2000, mouse mAb; clone DCS156; CST), cyclin D3 (1:5000, mouse mAb; clone DCS22; CST), phospho-MEK_1/2_ (1:5000, rabbit mAb; CST), phospho-ERK_1/2_ (1:5000, mouse mAb; BD Biosciences, San Jose, CA, USA), and GAPDH (1:50,000, mouse mAb; clone 6C5; Ambion/ Invitrogen; Carlsbad, CA, USA). These antibodies were applied in immunoreaction enhancer solution (Can Get Signal Solution 1; Toyobo, Osaka, Japan). The membrane was incubated for 16 h at 4 °C. The secondary antibody, horseradish peroxidase (HRP)-conjugated anti-mouse or anti-rabbit IgG (Chemicon International, Temecula, CA, USA), was diluted 1:20,000 in immunoreaction enhancer solution. The membrane was incubated for 1 h at room temperature, exposed to X-ray film (Fuji Film, Tokyo, Japan) using enhanced chemical luminescence, and Western blotted with Enhanced chemiluminescence (ECL) Prime detection reagent (GE Healthcare Bio-Sciences AB, Uppsala, Sweden). 1,4-Diamino-2,3-dicyano-1,4-bis (2-aminophenylthio) butadiene (U0126) (1 µg) (Calbiochem, San Diego, CA, USA), a synthetic inhibitor of MEK_1/2_ [44], was dissolved in 247 µL DMSO to create 10 mM stock solution. Cells were incubated with U0126 (10 µM) in RPMI-1640 with FBS for 2 h, added with MytiLec (100 µg/mL), and incubated for 12 h. Whole cell extracts were separated by SDS-PAGE on 12.5% gel and blotted onto PVDF membranes. Phosphorylated kinases were identified by western blotting with anti-phospho-MEK_1/2_, anti-MEK_1/2_, anti-phospho-ERK_1/2_, anti-ERK_1_, and HRP-conjugated anti-mouse IgG antibodies. Signals were detected by X-ray film exposure and western blotting as described above.

### 3.6. Statistical Analysis

Data are presented as mean ± SE. Differences between means were evaluated by two-tailed Student’s *t*-test, with *p* < 0.05 considered to be statistically significant.

## 4. Conclusions

We evaluated the mechanism of the cytotoxic effect of MytiLec purified from *Mytilus galloprovincialis* on Burkitt’s lymphoma Ramos cells. MytiLec triggered activation of various cell death pathways including MAPK cascade (phosphorylation of MEK-ERK, JNK, and p38 kinase pathways), death receptor signal (TNF-α expression), and activation of mitochondria-controlling caspase-9 and caspase-3, through interaction with glycan Gb3 present in glycosphingolipid-enriched microdomains on the cell surface.
